# Potential Protective Effect of Orlistat: A Formulation of Nanocrystals Targeting Inflammation, Oxidative Stress, and Apoptosis in an Experimental Model of Doxorubicin-Induced Cardiotoxicity

**DOI:** 10.3390/pharmaceutics16111356

**Published:** 2024-10-24

**Authors:** Maha Alsunbul, Thanaa A. El-Masry, Enas I. El Zahaby, Mohamed M. S. Gaballa, Maysa M. F. El-Nagar

**Affiliations:** 1 Department of Pharmaceutical Sciences, College of Pharmacy, Princess Nourah bint Abdulrahman University, P.O. Box 84428, Riyadh 11671, Saudi Arabia; maalsonbel@pnu.edu.sa; 2 Department of Pharmacology and Toxicology, Faculty of Pharmacy, Tanta University, Tanta 31527, Egypt; thanaa.elmasri@pharm.tanta.edu.eg; 3 Department of Pharmaceutics, Faculty of Pharmacy, Delta University for Science and Technology, Gamasa 35712, Egypt; elzahabi@deltauniv.edu.eg; 4 Department of Pathology, Faculty of Veterinary Medicine, Benha University, Toukh 13736, Egypt; mohamed.gaballah@fvtm.bu.edu.eg

**Keywords:** cardioprotective, orlistat nanocrystals, oxidative stress, inflammation, apoptosis, Sirt-1, Nrf2, HO-1

## Abstract

**Background**: Doxorubicin (DOX) is a widely used chemotherapeutic agent; nevertheless, cardiotoxicity limits its effectiveness. Orlistat (Orli) is an irreversible lipase enzyme inhibitor with poor solubility and bioavailability. Furthermore, Orli has a favorable impact on the decrease in cardiometabolic risk variables. Thus, this study aimed to investigate the novel use of Orlistat Nanocrystals (Orli-Nanocrystals) to mitigate DOX-induced cardiotoxicity and to identify probable pathways behind the cardioprotective effects. **Methods**: The pharmacokinetic parameters—area under % dose/g heart time curve (${AUC}_{0\to 4h}$), Drug targeting index (DTI), and relative targeting efficiency (RTE)—were calculated. Furthermore, experimental design mice were categorized into six groups: a (1) Normal control group, (2) Orli-Free group, (3) Orli-Nanocrystals group, (4) DOX group, (5) Orli-Free-DOX group, and (6) Orli-Nanocrystals-DOX group. All treatments were intraperitoneally injected once daily for 14 days with a single dose of DOX (15 mg/kg) on the 12th day for 4, 5, and 6 groups. **Results**: The pharmacokinetic parameters (C_max_, AUC) following oral administration of Orli-Nanocrystals presented a significant difference (higher values) in comparison to Orli due to the enhanced extent of the absorption of nanocrystals and, subsequently, their distribution to the heart. The study results indicated that DOX caused significant cardiotoxicity, as revealed by a remarkable rise in cardiac function biomarkers like LDH and CK-MB, which involve enzyme activities. Additionally, cardiac MDA content also increased; however, glutathione peroxidase, catalase, and superoxide dismutase activities were decreased. In the same context, DOX was found to have a remarkable downregulation in Nrf2, HO-1, Sirt-1, and Bcl2, while the upregulation of NF-κB, TNF-α, and BAX gene and protein expression occurred. Pretreatment with Orli-Nanocrystals displayed the most notable recovery of the altered immunohistochemical, histological, and biochemical characteristics as compared to the Orli-Free group. **Conclusions**: This work is the first investigation into the potential use of antioxidant, anti-inflammatory, and anti-apoptotic characteristics of Orli-Nanocrystals to protect against DOX-induced cardiotoxicity in vivo.

## 1. Introduction

A common anthracycline chemotherapeutic drug for the treatment of many tumors is doxorubicin (DOX), which has strong therapeutic benefits. All the same, cumulative and dose-dependent cardiotoxicity limits its clinical utility and may eventually cause irreversible structural abnormalities in the myocardium, as well as progressive heart failure [1]. Iron metabolism, altered Ca^2+^ balance, mitochondrial dysfunction, fibrosis, oxidative stress, endoplasmic reticulum stress, inflammatory response, apoptosis, and dysregulation of autophagy are among the complex and poorly understood pathophysiologies of DOX-induced cardiotoxicity [1,2].

Therefore, understanding the processes underlying DOX-induced cardiotoxicity is crucial to creating successful treatment plans. Notably, it has been established that DOX-induced cardiotoxicity is primarily influenced by oxidative stress and cardiac cell death [2]. Thus, two effective treatment approaches for DOX-induced cardiotoxicity are preserving cardiac redox equilibrium and reducing cell death.

As a result, several methods have been developed to promote cardioprotection while treating cancer. However, these medications’ have been found to have detrimental side effects and the loss of essential cardiac consequences in the years following the conclusion of therapy. There are no ideal treatments for DOX-induced cardiotoxicity, despite all recommended methods to lessen the cardiotoxicity of doxorubicin [3].

The most commonly given prescription medication for lowering body fat mass is orlistat (Orli). The concurrent lowering of cardiometabolic risk markers, including blood pressure, lipid profiles, the atherogenic index, insulin, and Homeostatic Model Assessment for Insulin Resistance (HOMA-IR) levels, has also been positively impacted by it [4]. Orli is a non-centrally acting anti-obesity medication that acts locally in the gastrointestinal tract to block lipase, an enzyme that is essential for the breakdown of long-chain triglycerides [5].

When compared to lifestyle changes alone, a number of randomized controlled trials have shown the clinical usefulness of orlistat in promoting weight loss [6,7]. Moreover, orlistat has additionally been shown to induce favorable alterations in several cardiovascular risk variables [8,9]. Despite this, no research has looked at its effects on cardiovascular events yet [10].

Due to its weakened chemical stability, low melting point, and waxy texture, orli is regarded as an unmanageable and troublesome product from a technological standpoint [11]. Nanocrystals are fine drug particles in the range of 1 to 1000 nm in size formulated through bottom-up technology; the main component is the drug, with the aid of a non-anionic surfactant to act as a stabilizer [12].

Nanocrystals are particularly beneficial for drugs like orlistat due to their simplicity, high drug-loading capacity, and stability. Unlike liposomes or polymeric nanoparticles, which may require complex preparation processes and include additional excipients, nanocrystals are composed purely of the drug itself. This minimizes formulation complexity and ensures that the maximum amount of drug reaches its target site [13,14].

Additionally, nanocrystals bypass the need for surfactants or solvents that can complicate the stability and safety profile of a formulation, making them an ideal choice for drugs that are intended for long-term use, such as Orli. These factors, combined with their ability to improve oral bioavailability, make nanocrystals a superior choice for the formulation of Orli over other nanoparticle systems [15,16].

Based on the Noyes–Whitney and Ostwald–Freundlich equations, the particle size at the nanoscale improves solubility. Furthermore, nanocrystals can rapidly permeate into the pore channels of the mucus layer lining the GIT and tightly bind to them, extending the effective range and duration of medicines in the gastrointestinal tract. More than 30% of all therapeutic product applications using nanomaterials are for nanocrystal-containing formulations [17].

Additional advantages of nanocrystals include the ability to improve drug metabolism behavior, hence minimizing harmful side effects and increasing patient compliance. As a result, pharmaceutical companies are becoming increasingly interested in nanocrystals’ outstanding characteristics [17].

The utilization of the radio-labeling technique with technetium-99m (99mTc) can be employed as an inexpensive accurate method for tracing orlistat in different body tissues, in addition to plasma [12].

For the first time, we hypothesize that Orli-Nanocrystals will attenuate DOX-induced cardiotoxicity in an experimental model of cardiotoxicity through anti-oxidant, anti-inflammatory, and anti-apoptotic mechanisms.

## 2. Materials and Methods

### 2.1. Drugs and Chemicals

The orlistat (Orli) was provided by Eva Pharma Co. (10th of Ramadan City, Egypt). El-Nasr Pharmaceutical Chemical Co., located in Obour, Egypt, supplied ethanol. A 99mMo/99mTc generator was provided by the Egyptian Atomic Energy Authority’s radioisotopes production facility (RPF), and the Egyptian Second Research Reactor (ETRR-2) was used to elute sodium pertechnetate (Na [99mTc] TcO4). Doxorubicin (Adricin^®^) was obtained from Hikma Pharmaceuticals, Cairo, Egypt. The other agents used were high-grade and analytical agents.

### 2.2. Preparation, Characterization, and Biodistribution Study of Orli-Nanocrystals

Orli-Nanocrystals were prepared; they were characterized via biodistribution study, as described by [12]. Orli-Nanocrystals were prepared using the solvent change method, utilizing Tween 80 as a stabilizer. The colloidal dispersion was allowed to freeze-dry, and particle size, zeta potential, PDI, DSC, XRD, and SEM examination were conducted; in addition, dissolution in 0.1 N HCl was conducted. In an evacuated vial, a mixture of 3 mg of Orli in 0.3 mL of DMSO was mixed with 2 mg of sodium borohydride (NaBH4) in pH 7 medium at room temperature, and 20 mL of a freshly eluted pertechnetate solution (99mTcO4−) (~60 MBq) was added to this combination. The reaction mixture was then maintained for 90 min [12].

A biodistribution study was authorized by the Egyptian Atomic Energy Authority’s Labelled Compounds Department’s animal ethics committee, and we followed the regulations established by Cairo University’s Faculty of Pharmacy (PT 4.2.2) and its animal ethics committee’s guidelines. Twenty-four mice were divided randomly into 4 subgroups representing time intervals (30, 60, 120, and 240 min). The percentage of orally administered dose/gram tissue was calculated in the following organs: blood, bone, muscle, brain, thyroid gland, liver, intestine, stomach, heart, lung, spleen, kidney, and urine [12].

### 2.3. Pharmacokinetic Study

The pharmacokinetic parameters included area under %dose/gram heart time curve (${AUC}_{0\to 4h}$), drug targeting index (DTI), and relative targeting efficiency (RTE), which were calculated according to the following equations [18,19,20,21]:

(1) $$({AUC}_{0\to 4h})=\sum \left(\frac{C_{n}+C_{n+1}}{2}\right)\times \left(t_{n+1}-t_{n}\right)$$

where

(${AUC}_{0\to 4h}$) is the area under the curve of the relation between the %Orli/g tissue and time from zero to 4 h.
C: represents the % Orli/g tissue at a certain time (0, 0.5, 1, 2, and 4 h).
T: represents the specific time (0, 0.5, 1, 2, and 4 h).

(2) $$\mathrm{DTE}=({AUC}_{0\to 4h})\;\mathrm{heart}/({AUC}_{0\to 4h})\;\mathrm{Blood}$$

where

(${AUC}_{0\to 4h}$) heart is the area under the curve of the relation between %dose of Orli/Orli-Nanocrystals/g heart versus time (h).
(${AUC}_{0\to 4h}$) Blood is the area under the curve of the relation between %dose of Orli/Orli-Nanocrystals/g Blood versus time (h).

(3) $$\mathrm{DTI}=\frac{\%\;dose\;of\;Orli-Nanocrystal/gheart\;\;}{\%\;dose\;of\;Orli/gheart\;}$$

where

The %dose of Orli-Nanocrystals/g Heart at a certain time is (0.5 to 4 h).
The %dose of Orli/g Heart at a certain time is (0.5 to 4 h).

(4) $$\mathrm{RTE}=\frac{{\left(AUC\right)}_{0\to 4\;Orli-Nanocrystals\;heart}}{{\left(AUC\right)}_{0\to 4\;orli\;heart}}$$

where

(${AUC}_{0\to 4h}$) heart NC is the calculated area under the curve of the relation between %dose of Orli-Nanocrystals/g heart versus time.
(${AUC}_{0\to 4h}$) heart Orli is the calculated area under the curve of the relation between %dose of Orli-/g heart versus time.

On the other hand, the maximum %dose/g heart (C_max_) was estimated directly from the biodistribution table, as well as the time corresponding to the maximum %dose/g heart (T_max_).

### 2.4. In Vivo Study

#### 2.4.1. Animals

Thirty-six male Swiss albino mice (12–15 weeks old), weighing between 22 and 25 g, were procured from the National Research Centre in Egypt. The animals had regular food and unfettered access to water. The study adhered to the organization’s guidelines for treating animals humanely. The study protocol has been accepted by the research ethics committee of the University of Tanta’s Faculty of Pharmacy. The Council for Medical Sciences (CIOMS) Code of Protocol (TP/RE/6/23p-0055) of the International Organisation was followed.

#### 2.4.2. Experimental Design

Six groups of mice, each counting 6 mice, were used: (1) Normal control group: mice were injected with vehicle; (2) Orli-Free group: mice were injected with Orli (240 mg/kg, I.P.) dissolved in 10% ethanol once daily for 14 days [22]; (3) Orli-Nanocrystals group: mice were injected intraperitoneal (I.P.) with Orli-Nanocrystals (240 mg/kg) dissolved in 10% ethanol once daily for 14 days [22]; (4) Doxorubicin (DOX) group: DOX was administrated as a single dose of (15 mg/kg, I.P.) on the 12th day [23] with some modifications; (5) Orli-Free-DOX group: mice were injected with Orli (240 mg/kg bd. wt., I.P.) dissolved in 10% ethanol once daily for 14 days, and a single dose of DOX (15 mg/kg, I.P.) on the 12th day; and (6) Orli-Nanocrystals-DOX group: mice were injected with Orli-Nanocrystals (240 mg/kg, I.P.) dissolved in 10% ethanol once daily for 14 days and a single dose of DOX (15 mg/kg, I.P.) on the 12th day.

#### 2.4.3. Samples Collection

On day 14, 48 h after receiving DOX, the mice were anesthetized with isoflurane, and blood samples were taken. Afterward, the mice were sacrificed by cervical dislocation, and the heart tissues were removed. The serum was divided by centrifugation (Sigma 2-16KL Osterode, Germany) and kept at 4 °C for 15 min at 4000 rpm to measure the cardiac enzymes. Part of the heart samples were preserved at room temperature in a 10% buffered formalin solution to be examined histopathologically and immunohistochemically. Furthermore, additional parts were kept at −80 °C until use. Subsequently, the kept tissue samples were homogenized in phosphate buffer saline (pH = 7.2) to assess different biochemical analyses.

#### 2.4.4. Cardiac Enzymes Determination

Serum samples were examined for lactate dehydrogenase (LDH) and creatine kinase MB (CK-MB) according to the manufacturer’s instructions for kits. The kits were brought from SPINREACT, Santa Coloma Bas, Spain, cat no. BEIS16-E and BEIS04-I, respectively.

#### 2.4.5. Oxidative Stress and Antioxidant Biomarkers Measurement

Lipid peroxidation (MDA) content, glutathione peroxidase (GPX-1), catalase (CAT), and superoxide dismutase (SOD) enzyme activity were evaluated in cardiac tissues using numerous commercial ELISA kits. They were purchased from MyBioSource.Co., San Diego, CA, USA, and CUSABIO.Co., Houston, TX, USA (Cat No: MBS268427, MBS9425463, MBS2600683, and CSB-E08555, respectively). They were assessed following the kit manufacturer’s instructions.

#### 2.4.6. Determination of TNF-α, HO-1, Sirt-1, and Nrf2 Content

TNF-α, HO-1, Sirt-1, and Nrf2 content were determined in cardiac tissues using ELISA kits obtained from CUSABIO.Co. Houston, TX, USA, and BT LAB. Co., Sessa Aurunca, Italy (Cat No. CSB-E04741, CSB-E08267, E1145, and E1083, respectively). It was assessed in compliance with the kit’s manufacturer’s guidelines.

#### 2.4.7. Quantitative Evaluation of Gene Expression of NF-κB, TNF-α, HO-1, Sirt-1, Nrf2, BAX, and Bcl2 Using Real-Time PCR (qRT-PCR)

By employing *β-actin* as a housekeeping gene in qRT-PCR, the relative gene expression of NF-κB, TNF-α, HO-1, Sirt-1, Nrf2, BAX, and Bcl2 was determined. Table 1 offers an assortment of primer sequences. The TRIzol reagent (15596026) from Life Technologies, Thermo Fisher Scientific, Colorado Springs, USA was used to extract total RNA.

The QuantiTects Reverse Transcription Kit (Qiagen, Hilden, Germany) was used to perform the reverse transcription procedure. Primers, complementary DNA amplicons, and Syber green master mix (Maxima SYBR Green/qPCR Master Mix, Thermo Fisher Scientific, Colorado Springs, USA) were included in the reaction mixtures. The gene expression was calculated using fold change techniques relative to the calibrator control group (2^−ΔΔCt^) [24].

**Table 1:** Primers sequence.

Gene	Primers Sequence (5′-3′)	Reference
***NF-κB***	GCAAACCTGGGAATACTTCATGTGACTAAG ATAGGCAAGGTCAGAATGCACCAGAAGTCC	[25]
***TNF-α***	CACCAGCTCTGAACAGATCATGA TCAGCCCATCTTCTTCCAGATGGT	[26]
***HO-1***	CTAAGACCGCCTTCCTGCTC GACGAAGTGACGCCATCTGT	[27]
***Sirt-1***	CAC-CAG-AAA-GAA-CTT-CAC-CAC-CAG ACC-ATC-AAG-CCG-CCT-ACT-AAT-CTG	[28]
***Nrf2***	AAGCAGCATAGAGCAGGACA GTTGCCCACTTCTTTTTCCA	[27]
***BAX***	CACCAGCTCTGAACAGATCATGA TCAGCCCATCTTCTTCCAGATGGT	[29]
***Bcl-2***	CACCCCTGGCATCTTCTCCTT AGCGTCTTCAGAGACAGCCAG	[29]
***β-actin***	GTG GGA ATT CGT CAG AAG GAC TCC TAT GTG GAA GTC TAG AGC AAC ATA GCA CAG CTT CTC	[30]

#### 2.4.8. Histopathological Examination

Four-micrometer-thick sections of heart tissue were carefully placed onto glass slides. The tissue sections underwent deparaffinization using xylene to remove the embedding medium, ensuring optimal tissue preparation for subsequent staining. Following deparaffinization, the sections were sequentially hydrated in ethanol solutions of descending concentrations: initially in 100% ethanol, followed by 95%, 80%, and finally 70% ethanol. Each immersion lasted 5 min per concentration step. This gradual rehydration process effectively replaced xylene with water, facilitating the subsequent histological staining procedures. The heart tissue sections were stained with Hematoxylin and Eosin (H&E) to assess the general histological architecture and identify any pathological changes. A light microscope with 400× magnification was used to conduct the histological study using the approach described by [31].

#### 2.4.9. Immunohistochemical Examination

To inactivate endogenous peroxidase, prepared atrial muscle paraffin slices were subjected to 3% H_2_O_2_, as per the instructions included in the immunohistochemical staining kit. Heat-induced antigen retrieval and serum-blocking techniques came next. The heart tissue slices were treated overnight at 4 °C with the relevant primary antibodies added, NF-κB (Santa-Cruz Biotechnology, Dallas, TX, USA Ca# sc-8008), Sirt-1 (Santa-Cruz Biotechnology, Dallas, TX, USA Ca# sc-74465), Bax (Invitrogen, Thermo Fisher Scientific, Colorado Springs, CO, USA, Cat# MA5-13-0300,), and Bcl-2 (Abcam, Cambridge, UK, Cat# ab182858). After adding and incubating a secondary antibody for half an hour, DAB and hematoxylin counterstaining were performed. Through a microscope, the stained slides were examined. The ratio of the positive area to the entire area was used to determine the positive expression, as explained by [32].

### 2.5. Statistical Analyses

The data were shown as mean ± S.D. To determine the differences between the groups, one-way ANOVA was followed by Tukey’s multiple comparisons. It was decided to use a *p*-value of less than 0.05 to indicate statistical significance. Statistical calculations were performed using GraphPad Prism, version 5 (GraphPad Software Inc., La Jolla, CA, USA). Additionally, the findings of the pharmacokinetic studies were computed using an unpaired *t*-test, in which a value is deemed significant if the *p*-value is less than 0.05.

## 3. Results

**Figure 1:**
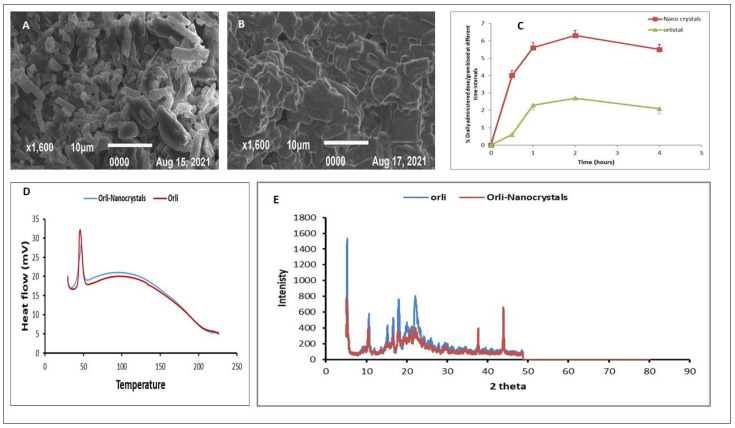
Summary of the physicochemical properties of Orli and Orli-Nanocrystals; SEM of Orli-Nanocrystals (**A**), SEM of Orli (**B**), %dose/g blood following oral administration of ^99mTc^Orli/^99mTc^ Orli-Nanocrystals to mice versus time (**C**), the DSC of Orli/Orli-Nanocrystals (**D**), and the XRD of Orli/Orli-nanocrystals (**E**), as adopted from ref. [12].

The Orli-Nanocrystals were fabricated utilizing Tween 80 as a stabilizer by solvent change method [12]. The physicochemical characteristics of Orli-Nanocrystals, such as particle size, zeta potential, polydispersity index (PDI), solubility in water, and 0.1 N HCl, were executed. The dissolution test in 0.1 N HCl was carried out at a speed of 75 rpm (37 °C), and the percentage dissolved after 60 min (%Q60) and percentage dissolution efficiency after 60 min were summarized (Table 2). DSC, XRD, and SEM techniques were performed (Figure 1).

### 3.1. Pharmacokinetic Analysis

**Figure 2:**
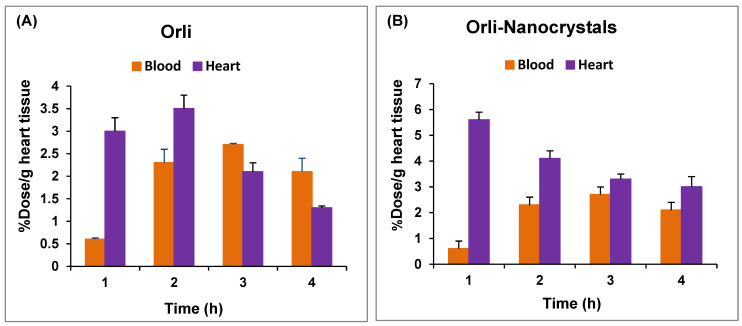
%Dose/g (heart tissue and blood) following oral administration to ^99mTc^Orli (**A**) and ^99mTc^Orli-Nanocrystals (**B**) (*n* = 3, mean ± SD).

The area under the % dose/gram heart tissue time relation calculated by the non-compartmental trapezoidal method showed higher values for the nanocrystals (13.73 ± 0.34 %dose/g heart. h) compared to the plain drug (8.52 ± 0.5 %dose/g heart. h); the RTE was 1.61 ± 0.06, while the DTE, on the contrary, was reduced (0.66 ± 0.033). The DTI was always higher than 1 for all time intervals, especially after 4 h (Table 3 and Figure 2A,B).

### 3.2. In Vivo Study

#### 3.2.1. Biochemical Markers

##### Effect of Orli-Nanocrystals on Cardiac Enzymes

As revealed in Table 4, the DOX group showed a significant increase in serum levels of LDH and CK-MB enzyme activities (5.47- and 6.85-fold, respectively) compared to the control group. Furthermore, the Orli-Free DOX group showed a significant decrease in the LDH and CK-MB levels (34.94% and 31.98%, respectively) in contrast to the DOX group. Also, the Orli-Nanocrystals DOX group showed a noteworthy reduction in the LDH and CK-MB cardiac enzyme levels (61.20% and 60.66%, respectively) compared to the DOX group. Moreover, the Orli-Nanocrystals DOX group displayed a significant decrease in the levels of LDH and CK-MB enzyme activities (40.35% and 42.17%, respectively) as compared to the Orli-Free DOX group (Table 4).

##### Effect of Orli-Nanocrystals on Oxidative Stress and Antioxidant Biomarkers in Cardiac Tissue

**Figure 3:**
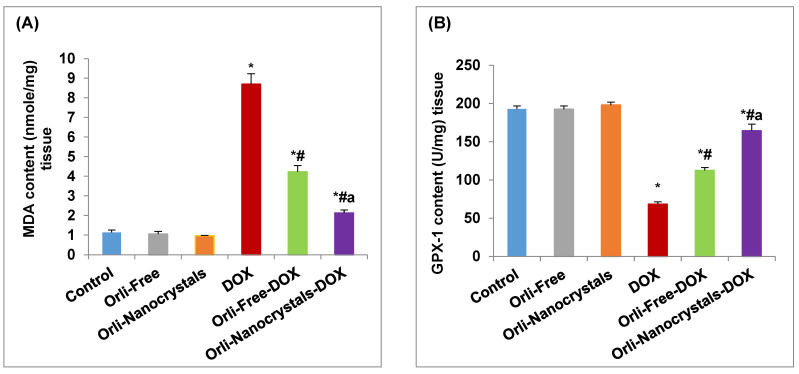

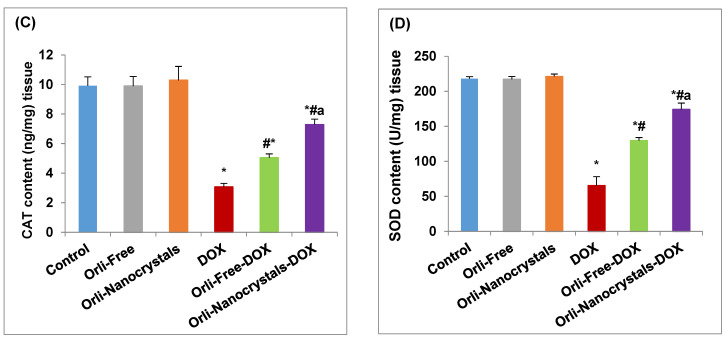
Effect of Orli-Nanocrystals on oxidative stress and antioxidant biomarkers in cardiac tissue. (**A**) MDA content, (**B**) GPX-1, (**C**) CAT, and (**D**) SOD enzyme activities. Data presented as mean ± SD (n = 6). * means significant vs. control group, ^#^ means significant vs. DOX group, ^a^ means significant vs. Orli-Free-DOX. DOX: Doxirubicin and Orli: Orlistat. Significant difference accepted at *p*-value ≤ 0.05.

As shown in Figure 3A, the DOX group showed a significant increase (6.62-fold) in MDA content compared to the control group. Conversely, though, the Orli-Free DOX and Orli-Nanocrystals DOX groups showed a significant decrease (51.32% and 75.50%, respectively) in MDA content compared to the DOX group. Moreover, the Orli-Nanocrystals DOX group presented a significant decrease (49.65%) in MDA content compared to the Orli-Free DOX group (Figure 3A).

In contrast, the GPX-1 enzyme activity in the DOX group significantly declined (64.53%) compared to the control group (Figure 3B). Nevertheless, the exhausted activity was returned in the Orli-Free DOX and Orli-Nanocrystals DOX groups (64.79% and 140.27%, respectively) compared to the DOX group. The Orli-Nanocrystals DOX group presented a noteworthy rise (45.8%) in enzyme activity compared to the Orli-Free-DOX group (Figure 3B).

In addition, the CAT enzyme activity in the DOX group was significantly reduced (69.02%) compared to the control group (Figure 3C). However, the depleted CAT enzyme activity was restored in the Orli-Free DOX and Orli-Nanocrystals DOX groups (64.17% and 137.46%, respectively) compared to the DOX group. Additionally, the Orli-Nanocrystals-DOX group displayed a significant increase (44.64%) in enzyme activity compared to the Orli-Free-DOX group (Figure 3C).

Likewise, the SOD enzyme activity in the DOX group was significantly reduced (69.99%) compared to the control group (Figure 3D). However, the depleted SOD enzyme activity was restored in the Orli-Free-DOX and Orli-Nanocrystals-DOX groups (98.03% and 165.94%, respectively) compared to the DOX group. Also, the Orli-Nanocrystals-DOX group revealed significantly amplified (34.29%) enzyme activity compared to the Orli-Free-DOX group (Figure 3D).

##### Effect of Orli-Nanocrystals on TNF-α, HO-1, and Nrf2 Content in Cardiac Tissue

**Figure 4:**
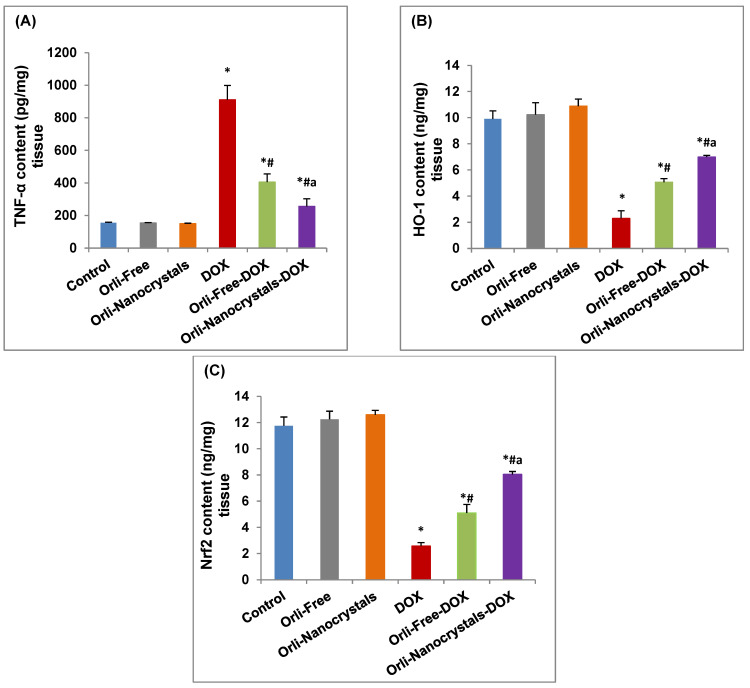
Effect of Orli-Nanocrystals on TNF-α, HO-1, and Nrf2 content in cardiac tissue. (**A**) TNF-α, (**B**) HO-1, and (**C**) Nrf2 content. Data presented as mean ± SD (*n* = 6). * means significant vs. control group, ^#^ means significant vs. DOX group, ^a^ means significant vs. Orli-Free-DOX. DOX: Doxirubicin and Orli: Orlistat. Significant difference accepted at *p*-value ≤ 0.05.

As revealed in Figure 4A, the DOX group disclosed a significant increase (4.88-fold) in TNF-α content in contrast to the control group. Conversely, however, the Orli-Free-DOX and Orli-Nanocrystals-DOX groups showed a significant decrease (55.52% and 71.89%, respectively) in TNF-α content compared to the DOX group. Moreover, the Orli-Nanocrystals-DOX group presented a significant decrease (36.8%) in TNF-α content compared to the Orli-Free-DOX group (Figure 4A).

In contrast, the HO-1 content in the DOX group was significantly reduced (76.77%) compared to the control group (Figure 4B). However, the exhausted content was returned in the Orli-Free-DOX and Orli-Nanocrystals-DOX groups (120% and 203.9%, respectively) compared to the DOX group. The Orli-Nanocrystals-DOX group showed a significant increase (38.14%) in HO-1 content compared to the Orli-Free-DOX group (Figure 4B).

Likewise, the Nrf2 content in the DOX group was significantly reduced (78.04%) compared to the control group (Figure 4C). However, the depleted Nrf2 content was restored in the Orli-Free-DOX and Orli-Nanocrystals-DOX groups (97.67% and 211.63%, respectively) compared to the DOX group. Also, the Orli-Nanocrystals-DOX group showed a significant increase (57.65%) in the Nrf2 content compared to the Orli-Free-DOX group (Figure 4C).

#### 3.2.2. mRNA Expression Markers

##### Effect of Orli-Nanocrystals on mRNA Expression of Inflammation Genes (*NF-κB* and *TNF-α*)

**Figure 5:**
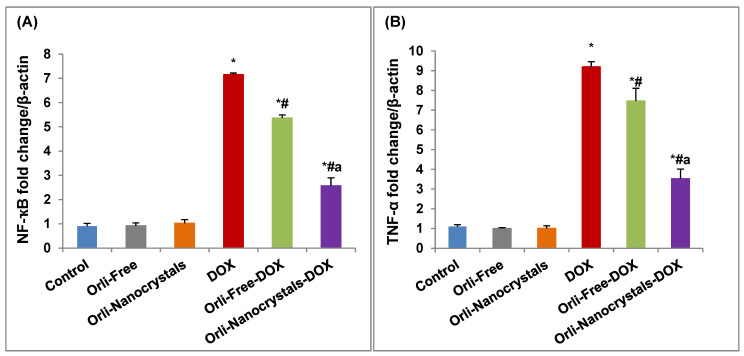
Effect of Orli-Nanocrystals on mRNA expression of inflammation genes (*NF-κB* and *TNF-α*). (**A**) *NF-κB* and (**B**) *TNF-α* mRNA expression. Data presented as mean ± SD (*n* = 3). * means significant vs. control group, ^#^ means significant vs. DOX group, ^a^ means significant vs. Orli-Free-DOX. DOX: Doxirubicin and Orli: Orlistat. Significant difference accepted at *p*-value ≤ 0.05.

As can be seen in Figure 5A, the DOX group displayed a significant upregulation (6.94-fold) in the cardiac levels of mRNA expression of *NF-κB* relative to the control group. Furthermore, the Orli-Free-DOX and Orli-Nanocrystals-DOX groups revealed a significant downregulation (24.9% and 64.06%, respectively) in the mRNA expression of *NF-κB* compared to the DOX group. Moreover, the Orli-Nanocrystals-DOX group presented an important downregulation (52.14%) in the cardiac levels of mRNA expression of *NF-κB* compared to the Orli-Free-DOX group (Figure 5A).

Likewise, the mRNA expression of *TNF-α* showed a significant upregulation (7.36-fold) in the cardiac levels of the mRNA expression of *TNF-α* relative to the control group (Figure 5B). Moreover, the Orli-Free-DOX and Orli-Nanocrystals-DOX groups revealed a significant downregulation (18.72% and 61.59%, respectively) in the mRNA expression of *TNF-α* compared to the DOX group. Moreover, the Orli-Nanocrystals-DOX group presented a significant downregulation (52.74%) in the cardiac levels of mRNA expression of *TNF-α* compared to the Orli-Free-DOX group (Figure 5B).

##### Effect of Orli-Nanocrystals on mRNA Expression of Apoptotic Genes (*BAX* and *Bcl-2*)

**Figure 6:**
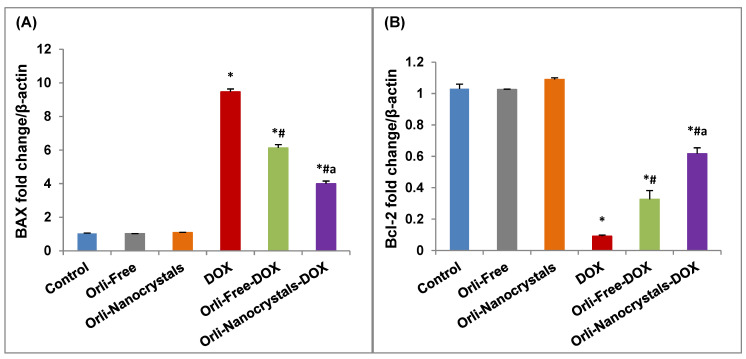
Effect of free and nanocrystals of orlistat on mRNA Expression of apoptotic genes (*BAX* and *Bcl-2*). (**A**) *BAX* and (**B**) *Bcl-2* mRNA expression. Data presented as mean ± SD (*n* = 3). * means significant vs. control group, ^#^ means significant vs. DOX group, ^a^ means significant vs. Orli-Free-DOX. DOX: Doxirubicin and Orli: Orlistat. Significant difference accepted at *p*-value ≤ 0.05.

As presented in Figure 6A, the DOX group exposed a noteworthy upregulation (8.19-fold) in the cardiac levels of mRNA expression of *BAX* relative to the control group. Furthermore, the Orli-Free-DOX and Orli-Nanocrystals-DOX groups revealed a significant downregulation (35.27% and 57.76%, respectively) in the mRNA expression of *BAX* compared to the DOX group. Moreover, the Orli-Nanocrystals-DOX group presented a substantial downregulation (34.75%) in the cardiac levels of mRNA expression of *BAX* compared to the Orli-Free-DOX group (Figure 6A).

In contrast, the mRNA expression of *Bcl-2* showed a significant downregulation (91.26%) in the cardiac levels of mRNA expression of *Bcl-2* relative to the control group (Figure 6B). Moreover, the Orli-Free-DOX and Orli-Nanocrystals-DOX groups revealed significant upregulation (2.67-fold and 5.89-fold, respectively) in mRNA expression of *Bcl-2* compared to the DOX group. Furthermore, mice pretreated with the Orli-Nanocrystals-DOX group presented a significant upregulation (87.88%) in the cardiac levels of mRNA expression of *Bcl-2* compared to the Orli-Free-DOX group (Figure 6B).

##### Effect of Orli-Nanocrystals on mRNA Expression of *Nrf2*, *HO-1*, and *Sirt-1* Genes

**Figure 7:**
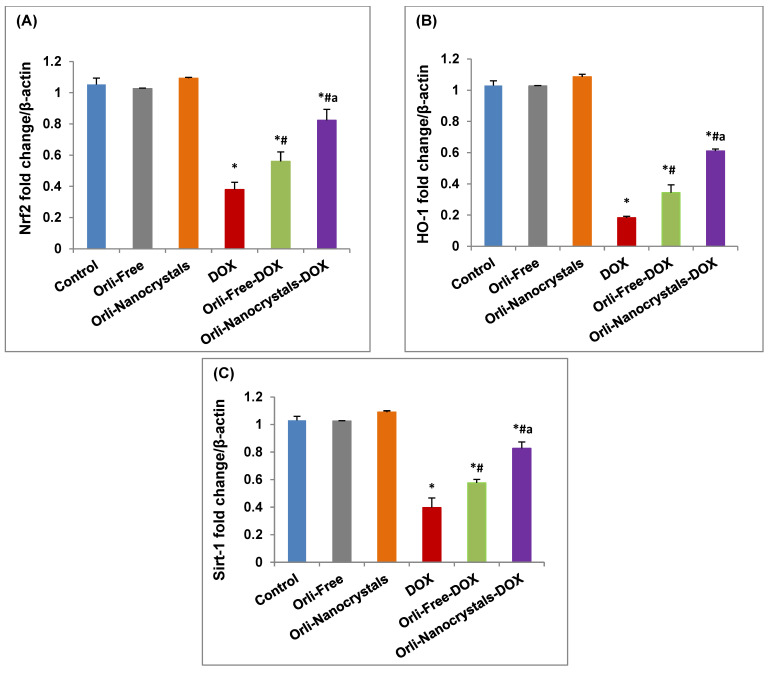
Effect of free and nanocrystals of orlistat on mRNA expression of *Nrf2*, *HO-1*, and *Sirt-1* genes. (**A**) *Nrf2*, (**B**) *HO-1*, and (**C**) *Sirt-1* mRNA expression. Data presented as mean ± SD (*n* = 3). * means significant vs. control group, ^#^ means significant vs. DOX group, ^a^ means significant vs. Orli-Free-DOX. DOX: Doxirubicin and Orli: Orlistat. Significant difference accepted at *p*-value ≤ 0.05.

As presented in Figure 7A, the mRNA expression of *Nrf2* showed a major downregulation (63.81%) in the cardiac levels of mRNA expression of *Nrf2* relative to a control group. Moreover, the Orli-Free-DOX and Orli-Nanocrystals-DOX groups revealed a significant upregulation (47.37% and 115.79%, respectively) in the mRNA expression of *Nrf2* compared to the DOX group. Moreover, mice pretreated with the Orli-Nanocrystals-DOX group presented a significant upregulation (46.43%) in the cardiac levels of mRNA expression of *Nrf2* compared to the Orli-Free-DOX group (Figure 7A).

Likewise, the mRNA expression of *HO-1* showed a significant downregulation (82.52%) in the cardiac levels of mRNA expression of *HO-1* relative to the control group (Figure 7B). Moreover, the Orli-Free-DOX and Orli-Nanocrystals-DOX groups revealed a significant upregulation (88.9% and 238.9%, respectively) in the mRNA expression of *HO-1* compared to the DOX group. Moreover, the Orli-Nanocrystals-DOX group presented a significant upregulation (79.41%) in the cardiac levels of mRNA expression of *HO-1* compared to the Orli-Free-DOX group (Figure 7B).

In the same manner, the mRNA expression of *Sirt-1* showed a considerable downregulation (61.17%) in the cardiac levels of mRNA expression of *Sirt-1* relative to the control group (Figure 7C). Moreover, the Orli-Free DOX and Orli-Nanocrystals DOX groups revealed a significant upregulation (42.5% and 108%, respectively) in the mRNA expression of *Sirt-1* compared to the DOX group. Moreover, the Orli-Nanocrystals-DOX group presented a significant upregulation (45.61%) in the cardiac levels of mRNA expression of *Sirt-1* compared to the Orli-Free-DOX group (Figure 7C).

#### 3.2.3. Histological and Immunohistochemical Analysis

##### Effect of Orli-Nanocrystals on Cardiac Histopathological Changes

**Figure 8:**
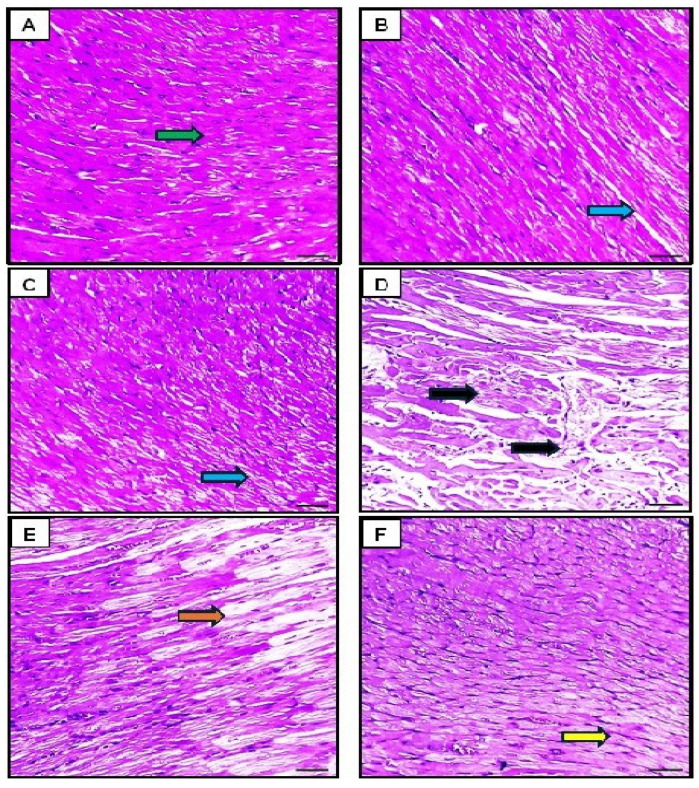
Histological examination of cardiac tissues revealed distinct characteristics across the groups: (**A**) Control group showed normal cardiac architecture with well-organized myocardial fibers and no signs of damage. (**B**) Orli-Free group appeared similar to the control group, with mild widening but no significant damage. (**C**) Orli-Nanocrystals group displayed normal cardiac muscle fibers with mild, non-significant vacuolation. (**D**) DOX group exhibited significant myocardial damage, including cellular necrosis, interstitial edema, and myofibril disarrangement. (**E**) Orli-Free-DOX group showed moderate myocardial damage with improved structure and preserved nuclei compared to the DOX group alone. (**F**) Orli-Nanocrystals-DOX group had nearly normal cardiac tissue with substantially reduced damage and mild histopathological changes. Green arrows indicate normal tissue, blue arrows indicate insignificant changes, yellow arrows indicate mild changes, orange arrows indicate moderate changes and black arrows indicate severe changes. Hematoxylin and eosin (H&E). (400×, Scale bar = 50 µm). DOX: Doxirubicin and Orli: Orlistat.

Upon histological examination, the control group showed a normal cardiac architecture with well-organized myocardial fibers. The cardiac muscle fibers exhibited a regular arrangement, intact cell and nuclear membranes, normal nuclear patterns, and no vacuolation. There was no evidence of cellular damage, inflammation, or fibrosis (Figure 8A). Cardiac tissue from the Orli-Free group appeared largely similar to that of the control group, with minimal histopathological changes. No significant damage or inflammation was observed. The tissues displayed a normal oval nucleus located peripherally and branching striated muscle fibers (Figure 8B). Also, the Orli-Nanocrystals group showed cardiac muscle fibers of normal shape, size, and configuration comparable to the control group. Mild interstitial edema and scattered widening of myofibrils were noted, but no significant necrosis or fibrosis was present (Figure 8C). In contrast, The DOX group showed significant myocardial damage with histological findings including cellular necrosis, loss of myocardial fibers, and marked interstitial edema. The cardiac muscle fibers exhibited perinuclear vacuolation, highly eosinophilic cytoplasm, widening of the myofibrils, and disarrangement and degeneration of the myocardium (Figure 8D). The group that received Orli-Free in addition to DOX exhibited moderate myocardial damage with a decrease in the number of degenerated myocardial fibers. The extent of damage was lower than that observed in the DOX-only group. The histological findings indicated improved myocardial structure with preserved nuclei and no fragmentation of the muscle fibers (Figure 8E). Likewise, the group treated with Orli-Nanocrystals showed almost normal cardiac muscle tissue with reduced myocardial damage compared to the group treated with DOX. Mild histopathological findings included slight interstitial edema and slight disarrangement of the muscle fibers (Figure 8F).

##### Effect of Orli-Nanocrystals on Cardiac NF-κB, BAX, Bcl2, and Sirt-1 Immunoexpression Levels

**Figure 9:**
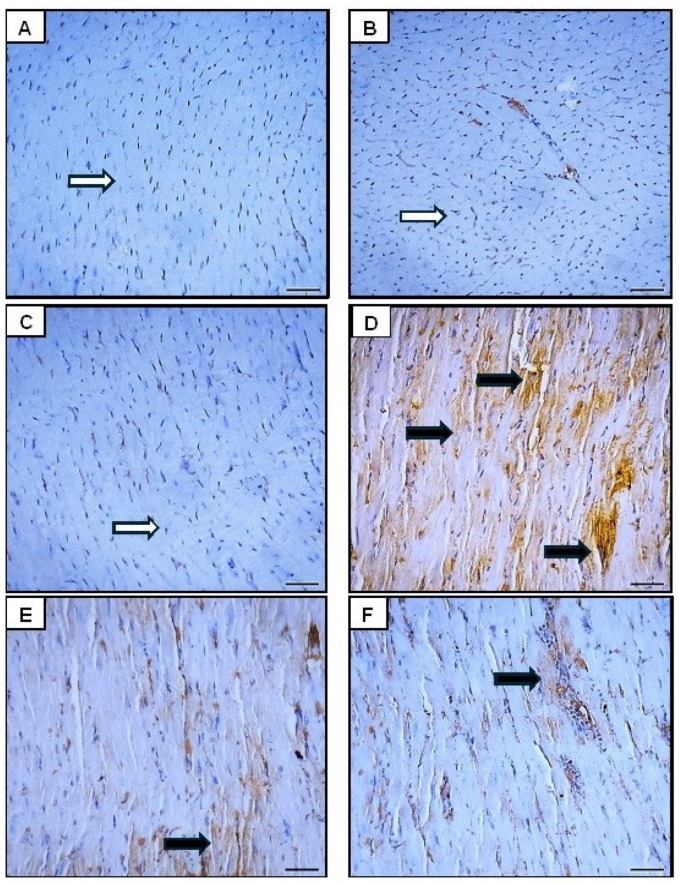

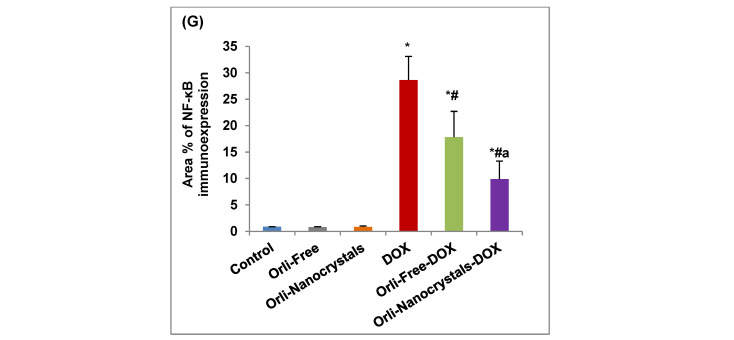
Photomicrographs showing NF-κB immunoreactivity in the sarcoplasm of cardiomyocytes across different groups (400×; Scale bar = 50 µm). (**A**) The control group section displayed no NF-κB immunoreactivity (white arrows). (**B**) Orli-Free group section exhibited minimal NF-κB immunoreactivity(white arrows) similar to the control group. (**C**) Orli-Nanocrystals group section showed negligible NF-κB immunoreactivity (indicated by white arrows) akin to the control group. (**D**) DOX group section demonstrated significantly elevated NF-κB immunoreactivity (indicated by black arrows), suggesting increased inflammatory signaling. (**E**) Orli-Free-DOX group section presented moderate NF-κB immunoreactivity (indicated by black arrows), indicating a reduced inflammatory response compared to the DOX group alone. (**F**) Orli-Nanocrystals-DOX group section displayed a decrease in NF-κB immunoreactivity (indicated by black arrows) compared to the DOX group, suggesting further attenuation of the inflammatory response. (**G**) Area % of NF-κB immunoexpression. Data presented as mean ± SD (n = 6). * means significant vs. control group, ^#^ means significant vs. DOX group, ^a^ means significant vs. Orli-Free-DOX. DOX: Doxirubicin and Orli: Orlistat. Significant difference accepted at *p*-value ≤ 0.05.

**Figure 10:**
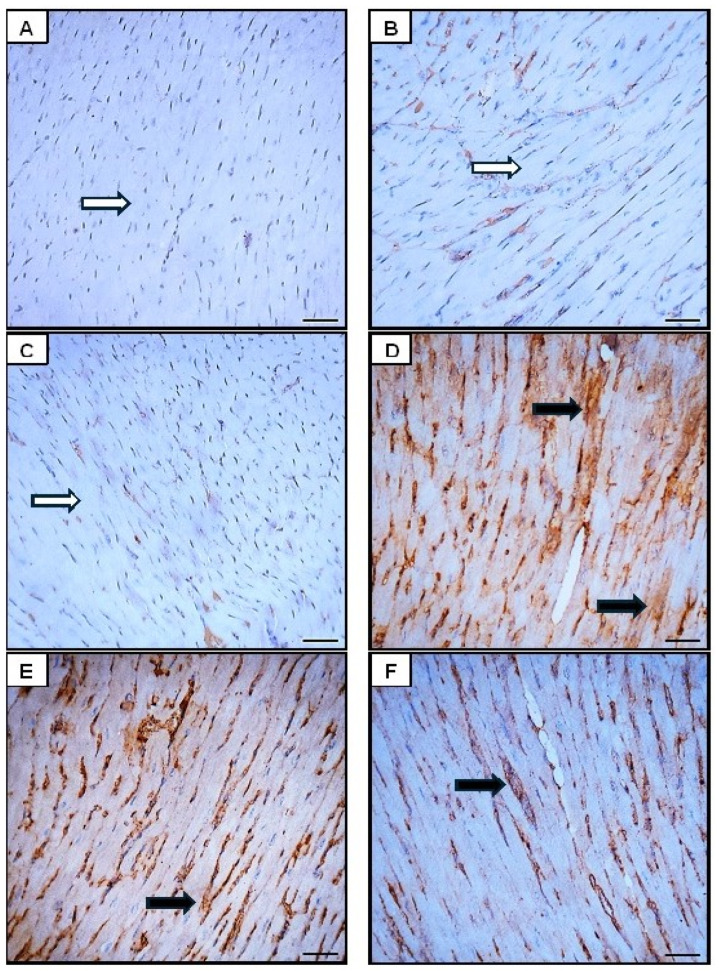

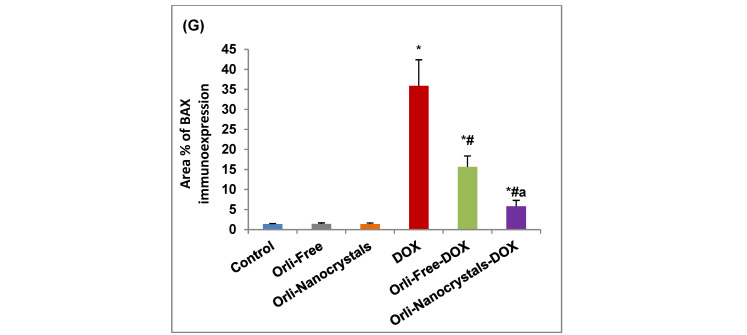
Photomicrographs showing BAX immunoreactivity in the sarcoplasm of cardiomyocytes across different groups (400×; Scale bar = 50 µm). (**A**) The control group section displayed minimal BAX immunoreactivity (white arrows) observed. (**B**) Orli-Free group section exhibited minimal BAX immunoreactivity (white arrows) comparable to the control group. (**C**) Orli-Nanocrystals group section showed negligible NF-κB immunoreactivity (indicated by white arrows) akin to the control group. (**D**) DOX group section demonstrated Low BAX immunoreactivity (white arrows) similar to the control group. (**E**) Orli-Free-DOX group section presented Moderate BAX immunoreactivity (black arrows), indicating reduced pro-apoptotic response compared to the doxorubicin group alone. (**F**) Orli-Nanocrystals-DOX group section displayed a decrease in BAX immunoreactivity (black arrows) compared to the DOX group, suggesting further attenuation of the pro-apoptotic response. (**G**) Area % of BAX immunoexpression. Data presented as mean ± SD (*n* = 6). * means significant vs. control group, ^#^ means significant vs. DOX group, ^a^ means significant vs. Orli-Free-DOX. DOX: Doxirubicin and Orli: Orlistat. Significant difference accepted at *p*-value ≤ 0.05.

**Figure 11:**
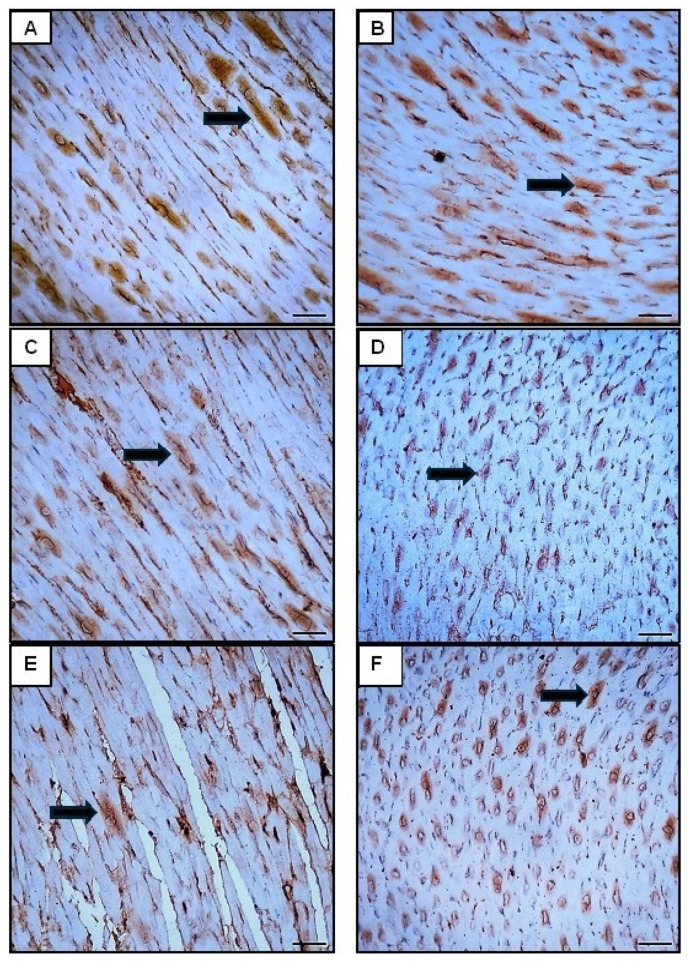

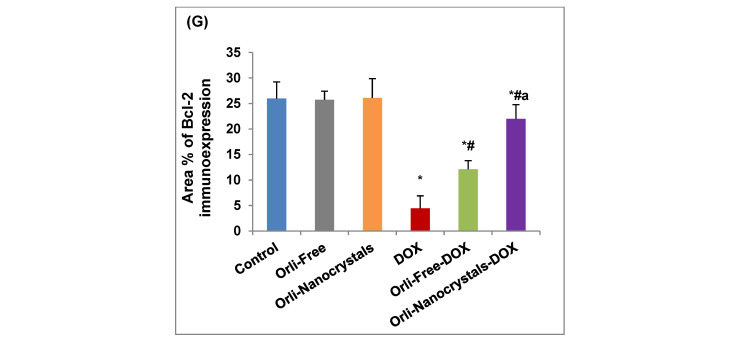
Photomicrographs showing Bcl-2 immunoreactivity in the sarcoplasm of cardiomyocytes across different groups (400×; Scale bar = 50 µm). (**A**) The control group section displayed high Bcl-2 immunoreactivity (black arrows), indicating strong anti-apoptotic signaling. (**B**) Orli-Free group section exhibited high Bcl-2 immunoreactivity, comparable to the control group. (**C**) Orli-Nanocrystals group section showed high Bcl-2 immunoreactivity comparable to the control group. (**D**) DOX group section demonstrated a decrease in Bcl-2 immunoreactivity, suggesting reduced anti-apoptotic signaling. (**E**) Orli-Free-DOX group section presented moderate Bcl-2 immunoreactivity (black arrows), showing improved anti-apoptotic signaling compared to the DOX group. (**F**) Orli-Nanocrystals-DOX group section showed an increase in Bcl-2 immunoreactivity (black arrows), indicating further improved anti-apoptotic signaling. (**G**) Area % of Bcl-2 immunoexpression. Data presented as mean ± SD (n = 6). * means significant vs. control group, ^#^ means significant vs. DOX group, ^a^ means significant vs. Orli-Free-DOX. DOX: Doxirubicin and Orli: Orlistat. Significant difference accepted at *p*-value ≤ 0.05.

The analysis of NF-κB, Bax, and Bcl-2 (Figure 9, Figure 10 and Figure 11) immunohistochemical staining revealed distinct differences among the groups. In the control group, minimal to no expression of NF-κB and Bax was observed, indicating no significant activation of the inflammatory pathway or pro-apoptotic signaling, while high levels of Bcl-2 expression indicated strong anti-apoptotic signaling and cell survival. The DOX group exhibited markedly increased expression of NF-κB and Bax, suggesting significant activation of the inflammatory response and enhanced pro-apoptotic signaling due to DOX administration. This group also showed significantly decreased Bcl-2 expression, indicating reduced anti-apoptotic signaling and increased susceptibility to cell death (Figure 8, Figure 9 and Figure 10).

The control group and the Orli-Free group exhibited minimal NF-κB and Bax expression, suggesting insignificant inflammatory activation or pro-apoptotic signaling. Additionally, high levels of Bcl-2 expression in both groups indicated strong anti-apoptotic signaling. Similarly, the Orli-Nanocrystals group displayed low levels of NF-κB and Bax expression, along with high levels of Bcl-2 expression, pointing to low inflammatory and pro-apoptotic activity, and strong anti-apoptotic signaling, comparable to the control group (Figure 9, Figure 10 and Figure 11).

The group treated with Orli-Free-DOX showed moderately increased the NF-κB and Bax expression, indicating a reduced inflammatory response and pro-apoptotic signaling compared to the DOX-only group. This group also exhibited moderately decreased Bcl-2 expression, suggesting improved anti-apoptotic signaling compared to the DOX-only group. The group treated with Orli-Nanocrystals-DOX displayed slightly increased NF-κB and Bax expression, further reducing the inflammatory response and pro-apoptotic signaling compared to the Orli-Free-DOX group. This group also showed slightly decreased Bcl-2 expression, indicating a further improvement in anti-apoptotic signaling compared to the Orli-Free-DOX group (Figure 9, Figure 10 and Figure 11).

**Figure 12:**
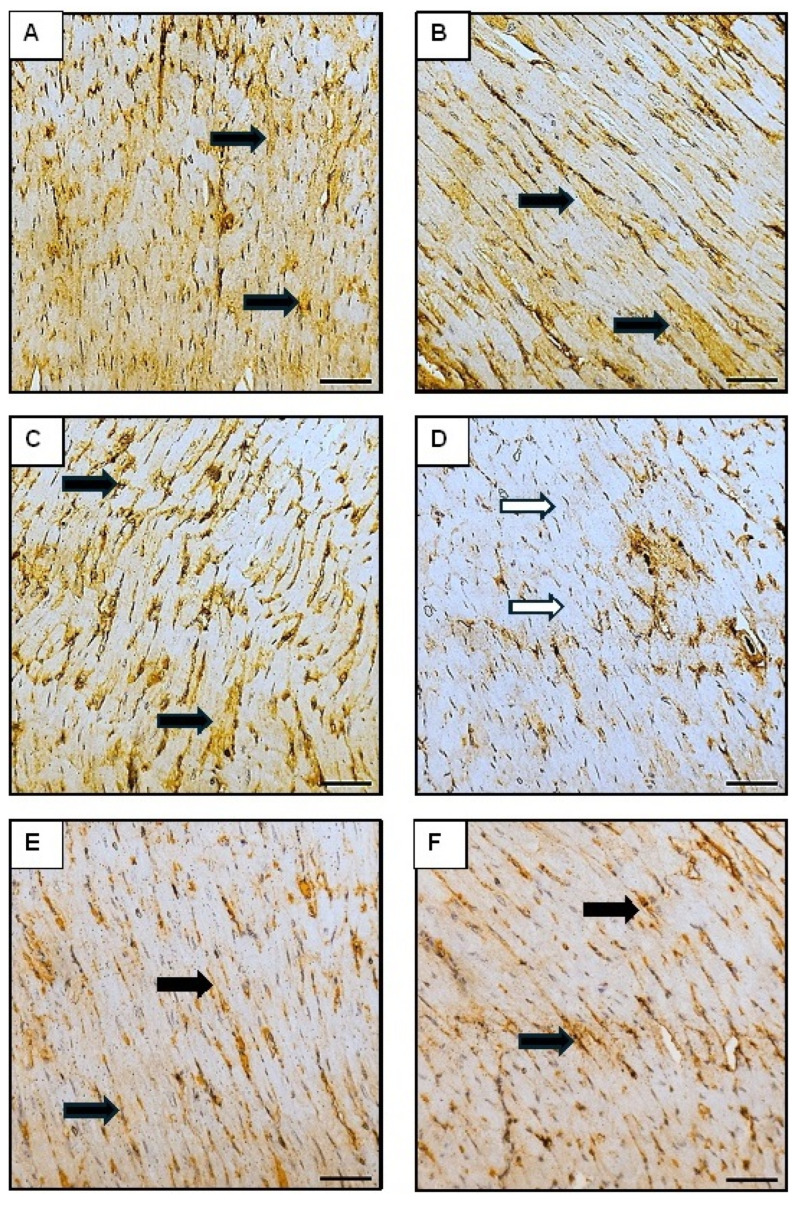

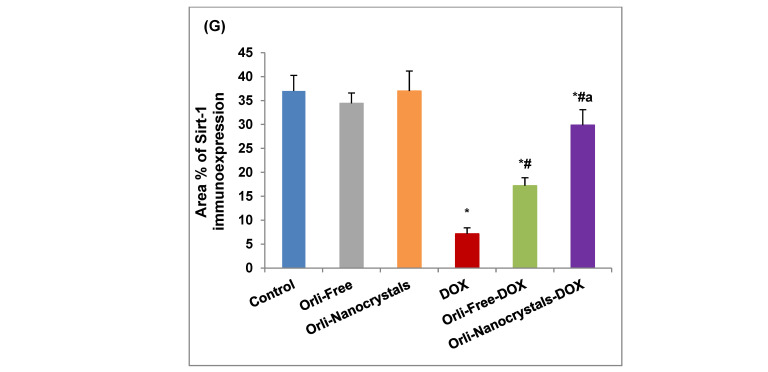
Photomicrographs showing Sirt-1 immunoreactivity in the sarcoplasm of cardiomyocytes across different groups (400×; Scale bar = 50 µm). (**A**) The control group displayed high Sirt-1 immunoreactivity (black arrows). Both (**B**) Orli-Free and (**C**) Orli-Nanocrystals groups showed also high Sirt-1 immunoreactivity (black arrows). (**D**) DOX group exhibited markedly decreased Sirt-1 immunoreactivity (white arrows), reflecting diminished cellular resilience. (**E**) Orli-Free-DOX group section demonstrated moderate Sirt-1 immunoreactivity (black arrows), indicating an improvement in expression and cellular resilience compared to the DOX group alone. (**F**) Orli-Nanocrystals-DOX group section showed further enhanced Sirt-1 expression (black arrows) and cellular resilience. (**G**) Area % of Sirt-1 immunoexpression. Data presented as mean ± SD (*n* = 6). * means significant vs. control group, ^#^ means significant vs. DOX group, ^a^ means significant vs. Orli-Free-DOX. DOX: Doxirubicin and Orli: Orlistat. Significant difference accepted at *p*-value ≤ 0.05.

For Sirt-1, the control group exhibited the highest average positive area ratio indicating strong expression associated with enhanced cellular survival and stress resistance (Figure 12). The DOX group had the lowest average positive area ratio, reflecting significantly diminished Sirt-1 expression. The Orli-Free group displayed a high average positive area ratio that was similar to the control group, indicating robust Sirt-1 expression. Similarly, the Orli-Nanocrystals group had a high average positive area ratio that was also comparable to the control group. The Orli-Free-DOX group showed an intermediate average positive area ratio, indicating improved Sirt-1 expression compared to the DOX group alone. The Orli-Nanocrystals DOX group exhibited an intermediate average positive area ratio, showing further enhancement in Sirt-1 expression and cellular resilience compared to the DOX and Orli-Free-DOX groups (Figure 12).

## 4. Discussion

DOX is an anticancer agent commonly used to treat malignant hematological and solid tumors in both adults and children. Its function is dependent on topoisomerase inhibition to slow cancer progression. However, DOX can also impact other organs in the body, and following chemotherapy, cancer patients’ life quality suffers as a result of the side effects [32,33]. Numerous studies have shown that DOX causes cardiotoxicity and cardiomyopathy via a variety of pathways, including reactive oxygen species (ROS) production and oxidative stress-related damage, cardiomyocyte apoptotic cell death, intracellular calcium disturbance, and impaired cardiac energy homeostasis. DOX is known to stimulate both the extrinsic and intrinsic apoptotic pathways. As a result, blocking apoptosis may help prevent DOX’s cardiotoxic effects [34].

Lipid-based carriers, polymeric carriers, metal nanoparticles, and carbon-structured and mineral particles are examples of nanomaterials utilized in medicine. Several tactics have been employed to improve the efficacy and safety profile of DOX, including the use of liposomes [33,35]. Liposomes were the first nano drug delivery method used in clinical trials, and the first approved drug nanocarrier was the PEGylated liposomal version of doxorubicin marketed as Doxil [36]. Their capacity to package both hydrophilic and hydrophobic medicines, as well as their biocompatibility and biodegradability, make them effective drug delivery carriers [36].

Liposomal formulations are preferred because they tend to sequester the medicine away from organs such as the heart, with higher concentrations in the liver, spleen, and tumors. However, the liposomal forms of doxorubicin are not without their own set of toxicities, which, in the case of Doxil, may be accompanied by the distinct toxicity of palmar-plantar erythrodysesthesia [37].

Patients with AIDS-related Kaposi’s sarcoma (ARKS), as well as a range of solid tumors, including ovarian, breast, and prostate carcinomas, have been studied in clinical trials with pegylated liposomal doxorubicin. The pharmacokinetic profile in humans at dosages ranging from 10 to 80 mg/m^2^ is comparable to that of animals, with one or two distribution phases: an initial phase with a half-life of 1–3 h and a second phase with a half-life of 30–90 h. The AUC following a dosage of 50 mg/m^2^ is roughly 300 times larger than that of a free drug. The clearance and distribution volume are dramatically reduced (at least 250-fold and 60-fold, respectively) [38].

Despite improvements in safety features, liposomal encapsulated DOX is not more effective than ordinary DOX. Functionalized (targeted) liposomes provide a more effective method for delivering DOX to the tumor. Furthermore, encapsulating DOX in pH-sensitive liposomes (PSLs) or thermosensitive liposomes (TSLs) and heating them locally increased DOX accumulation in the tumor [39].

In terms of medication delivery, many nanocarriers use the enhanced permeability and retention (EPR) effect to reach the appropriate target site via blood extravasation. The EPR effect is caused by aberrant or excessively permeable vasculature at locations of injury or inflammation. Several preclinical investigations have indicated the effectiveness of passive targeting of the cardiac myocardium after an infarction, enhancing heart functioning [35,40,41].

For instance, curcumin may help to reduce the cardiotoxic effects of DOX. The nano-formulation of curcumin, in particular, can overcome curcumin’s low bioavailability and improve its physicochemical qualities in terms of efficacy [42]. Also, apigenin-coated gold nanoparticles (Api-AuNPs) therapy resulted in significantly less body and cardiac weight loss than the DOX group, significantly reduced injury markers, and prevented myocardial apoptosis via regulating BAX, caspase3, and Bcl-2 while also reducing DOX-induced tissue damage [34].

Based on the previous studies, we have chosen Orli, which, has concurrently demonstrated a positive effect on the reduction of risk factors associated with cardiometabolic diseases, such as insulin, blood pressure, lipid profiles, and the atherogenic index [43,44,45,46].

Unfortunately, Orli is technically very difficult to formulate due to its waxy nature. Nanocrystals were a successful technique for improving its aqueous solubility, as well as the extent of absorption. Furthermore, nanocrystals can rapidly permeate into the pore channels of the mucus layer lining the GIT and tightly bind to them, extending the effective range and duration of medicines in the gastrointestinal tract [13,47].

Nanocrystals have several advantages over the other methods such as higher drug loading, enhanced solubility and bioavailability, reduced dosage frequency, and enhanced therapeutic effect, in addition to their stability [13,47,48].

The formulation of nanocrystals is a simple, inexpensive method contrary to other methods for the production of nanoparticles. For manufacturing nanoemulsion, stabilizing nanodroplets requires high concentrations of surfactants and co-surfactants. Nanoemulsions are expensive to create due to the smaller size of the droplets, which necessitates specialized equipment and processes. Creating a homogenizer for nanoemulsions can be pricey. Additionally, the microfluidization and ultra-sonication production processes require significant funding. The storage of nanoemulsion formulations is a key concern. Nanoemulsions can be pricey to distribute [49].

Radiolabeling with technetium-99m was considered a relatively low-cost method in comparison to other analytical techniques (such as LC Ms/Ms analytical method) to investigate the drug concentration in biological tissues. Radiolabeling has allowed the study of nanocrystals and plain drug distribution behavior to all body tissues via the calculation of the % of orally administered dose/gram tissue [12]. Based on the data of the former study by [12], the % dose of technetium Orli and technetium Orli Nanocrystals distributed to the heart was investigated.

Consequently, the pharmacokinetic parameters (C_max_, AUC) showed a significant difference (higher values) due to the enhanced extent of oral absorption of nanocrystals and, subsequently, their distribution to the heart. The % DTI (the partitioning time average of the ^99mTc^ orlistat and ^99mTc^orlistat nanocrystals between heart and blood) showed a significant increase. On the contrary, the lower values for T_max_ can be explained by enhanced absorption from the intestine due to their transformation into crystals in the nanosize in addition to their rode shape [50].

The lower DTE of Orli-Nanocrystals in comparison to Orli could be explained by the significant increase in the % dose/g blood and, consequently, the ability of nanoformulation to distribute to different tissues effectively. The RTE was 1.61, indicating the higher levels of Orli-Nanocrystals distributed to heart tissues. The Biopharmaceutical Classification System (BCS) places orlistat in class II drugs due to its high permeability and low solubility. This means that solubility is the rate-determining step of its absorption and biodistribution [51]. Nanoparticles can successfully bind to their specific receptor, and they must be able to travel in the bloodstream while avoiding absorption by macrophages, especially in the reticuloendothelial system [50].

The IP route is used in preclinical studies for poorly absorbed or extensively metabolized drugs when given orally. The IP is simple to learn, rapid to administer, appropriate for chronic treatments and has a low-stress impact on laboratory animals [52].

The correlation between the pharmacokinetics of drugs administered orally and intraperitoneally (IP) largely depends on factors such as absorption rates, bioavailability, and the physiological routes of drug distribution. Considering orally administered drugs, absorption typically occurs through the gastrointestinal tract, involving first-pass metabolism, which can significantly reduce the bioavailability of the drug. In contrast, intraperitoneal administration bypasses the gastrointestinal tract, allowing drugs to be absorbed directly into systemic circulation through the peritoneum, leading to more rapid absorption, especially for small molecules. The peritoneum has a large surface area and a rich blood supply, facilitating efficient drug transport. However, larger molecules rely more on lymphatic transport after IP administration [52].

Pharmacokinetically, IP administration often results in higher peak plasma concentrations and faster onset compared to oral administration [53].

Once absorbed, drugs administered via either route distribute throughout the body similarly, but the rate and extent of distribution may vary depending on the formulation and the drug’s physicochemical properties [53].

Our previous study examined the pharmacokinetics of radiolabeled Orli/Orli-Nanocrystals following oral administration; the results indicated the superior absorption and tissue distribution of Orli-Nanocrystals over Orli. Since the current study was designed to continue for 14 days utilizing thirty-six male Swiss albino mice, the IP route was selected instead of the oral route to decrease the dosing burden on the animals.

DOX-induced cardiotoxicity, involves several pathways, such as oxidant stress, iron metabolism, disruption of Ca^2+^ homeostasis, gene expression change, apoptosis, alteration, and other causes [51,54]. The present study suggests that Orlistat-Nanocrystals could restrict DOX cardiotoxicity via the renovation of genes NF-κB, TNF-α, HO-1, Sirt-1, Nrf2, BAX, and Bcl2, as well as the reduction in oxidative stress, inflammation, and apoptosis.

The results of this study demonstrated that the cardiotoxicity was caused by the DOX dosage (15 mg/kg, I.P., once), which was confirmed by an increase in the serum levels of LDH and CK-MB. These results are in line with earlier studies [55,56,57]. Additionally, DOX treatment caused remarkable cellular changes, as well as increased ROS and lipid peroxidation. Moreover, GPx-1, CAT, and SOD enzyme activities were significantly declined. These results monitored those described by [58,59,60]. In the same context, DOX (15 mg/kg, I.P., once) has a remarkable downregulation in Nrf2, HO-1, Sirt-1, and Bcl2, while it induces the upregulation of NF-κB, TNF-α, and BAX gene and protein expression. These outcomes followed those stated by [55,56,57,61,62].

Recent data have indicated that the main mediators of DOX-related cardiotoxicity are oxidative stress and the inflammatory response [63]. One electron was removed from quinone to quickly convert DOX to a semiquinone. Semiquinone autoxidized, resulting in the generation of superoxide anions [64,65]. The overproduction of ROS led to oxidative damage to proteins and lipids, which in turn caused heart dysfunction. This study suggests that pretreating animals with Orli-Free and Orli-Nanocrystals minimized DOX-induced ROS production and oxidative damage in cardiac tissues, with Orli-Nanocrystals’ greater protective impact being particularly notable. More concerning was the fact that DOX dramatically lowered the body’s natural antioxidant, making the heart more susceptible to oxidative stimuli brought on by DOX. In this regard, antioxidant system repair through Orli-Nanocrystals therapy might potentially help against cardiotoxic agents.

Hundreds of genes involved in cell defense against oxidative stress—the hallmark of many illnesses like neurodegenerative, cardiovascular, certain viral pathologies, diabetes, and others—are controlled by the Nrf2 transcription factor. The primary pathway by which Nrf2 activity is regulated is through interactions with the protein Keap1 [66]. In normal conditions, Nrf2 is bound by Keap1 and targeted for proteasomal degradation, allowing Keap1 to regenerate, while in oxidative stress conditions, the interactions between Nrf2 and Keap1 are disrupted, and Nrf2 initiates the transcription of protective genes. Now, activating the Nrf2 system is thought to be a potent cytoprotective approach for treating various diseases [67,68]. Among the several downstream enzymes that Nrf2 comprises are heme oxygenase-1 (HO-1), glutathione peroxidase, glutathione-S-transferase, and superoxide dismutase [67]. HO-1 is produced in response to inflammation and oxidative stress, shielding tissues from harm [67].

A common leucine zipper (bZIP) is an Nrf2 protein linked to DOX-induced cardiomyopathy according to reports. Nrf2 deficiency exacerbated the cardiotoxicity and cardiac effects of DOX malfunction in rodents [69]. On the other hand, in mice, Nrf2 activation with a pharmaceutical agent increased the production of antioxidant and antielectrophile enzymes, protecting against DOX toxicity [70].

Moreover, TNF-α transcription can be triggered by active NF-κβ, and TNF-α is eventually recognized to contribute to the pathophysiology of both acute and chronic inflammation [70]. Furthermore, according to earlier research, oxidative stress increases can trigger BAX, decrease Bcl-2 mRNA expressions, and impair mitochondrial function, which in turn activates the caspase pro-apoptotic pathway (Casp-8, Casp-9, and Casp-3) [71].

Sirtuin 1 (SIRT1) is a deacetylase that is reliant on nicotinamide adenine dinucleotide (NAD+) and regulates many cellular fates, including senescence, metabolic balance, and cardiomyocyte survival [72,73,74]. According to a clinical investigation, cardiac SIRT1 was downregulated (54.92% 7.80%) in samples of progressive heart failure in contrast to hearts in healthy control, which may have an impact on the rise in pro-apoptotic molecules and fall in antioxidants [75]. Furthermore, Sirt1 may prevent NF-κB activation, whereby is the primary regulator of the synthesis of pro-inflammatory cytokines. It may also regulate P53 and caspase-3 signaling, which are vital for the regulation of inflammation, oxidative stress, and apoptosis [76].

As far as we are aware, no research has looked into the protective effect of orlistat against DOX-induced cardiac toxicity. However, this study found that treating mice with Orli-Free and Orli-Nanocrystals could reduce DOX-induced cardiotoxicity by minimizing DOX-induced oxidative damage, inflammatory response, and apoptosis.

## 5. Conclusions

To the best of our knowledge, this work is the first to look at Orli-Nanocrystals’ possible defense against DOX-induced cardiotoxicity. In pharmacokinetic analysis, nanocrystals significantly improved the C_max_ and AUC, which are representative of enhanced cardiac distribution. The use of Orli-Nanocrystals had cardioprotective benefits that were confirmed by strong anti-inflammatory, anti-apoptotic, and antioxidant effects. Its potential application in DOX cardiotoxicity is supported by these cardioprotective qualities. The therapeutic uses of Orli-Nanocrystals in human patients should be investigated further in future studies.

## Figures and Tables

**Table 2:** Summary of the physicochemical properties of Orli-Nanocrystals and %dose/g heart following administration of [12].

Physicochemical Property				
Particle size (nm)			329.59 ± 120.75	
Zeta potential (mV)			−27.04	
PDI			0.277	
Aqueous solubility (µg/mL)			363.5 ± 43.37	
Solubility in 0.1 N HCl (µg/mL)			457.6 ± 90.35	
%Q60 (Mean ± SD, *n* = 3)			99.68.0 ± 19.43	
%DE60 min			66.18	
%dose/g heart				
Time	0.5	1	2	4
^99mTc^Orli	3.00 ± 0.30	3.50 ± 0.30 *	2.10 ± 0.20 *	1.30 ± 0.04 *
^99Tc^Orli-Nanocrystals	5.60 ± 0.30	4.10 ± 0.30 *	3.30 ± 0.20 *	3.00 ± 0.40 *

* Significant difference accepted at *p*-value ≤ 0.05.

**Table 3:** The pharmacokinetic parameters following the administration to ^99mTc^Orli and ^99mTc^Orli-Nanocrystals (*n* = 3, mean ± SD).

	C_max_ (%dose/g Heart Tissue)	T_max_ (h)	$({AUC}_{0\to 4h}$) (%dose/g Heart Tissue. h)	$\left({AUC}_{0\to 4h}\right)$ (%dose/g Blood. h)	DTE	RTE
^99mTc^Orli	3.5 ± 0.3	1	8.52 ± 0.5	8.13 ± 0.3	1.05 ± 0.02	1.61 ± 0.06
^99mTc^Orli- Nanocrystals	5.6 ± 0.3 *	0.5	13.73 ± 0.34 *	20.85 ± 0.75	0.66 ± 0.03 *	
**DTI**						
**Time (h)**			0.5	1	2	4
			1.82 ± 0.11	1.18 ± 0.014	1.66 ± 0.12	2.30 ± 0.2

* Significant difference accepted at *p*-value ≤ 0.05.

**Table 4:** Effect of Orli-Nanocrystals on cardiac enzymes.

Group	LDH (U/L)	CK-MB (U/L)
Control	141.73 ± 3.62	89.93 ± 0.83
Orli-Free	143.11 ± 4.64	88.98 ± 0.71
Orli-Nanocrystals	143.33 ± 2.98	88.14 ± 0.76
DOX	916.69 ± 63.55 *	705.81 ± 19.39 *
Orli-Free-DOX	596.41 ± 14.41 *^#^	480.09 ± 5.63 *^#^
Orli-Nanocrystals-DOX	355.7 ± 321.42 *^#a^	277.66 ± 5.43 *^#a^

Data presented as mean ± SD (*n* = 6). * means significant vs. control group, ^#^ means significant vs. DOX group, ^a^ means significant vs. Orli-Free-DOX. DOX: Doxirubicin and Orli: Orlistat. Significant difference accepted at *p*-value ≤ 0.05.

## Data Availability

The data are contained within the article.
